# Prp4 Kinase Grants the License to Splice: Control of Weak Splice Sites during Spliceosome Activation

**DOI:** 10.1371/journal.pgen.1005768

**Published:** 2016-01-05

**Authors:** Daniela Eckert, Nicole Andrée, Aleh Razanau, Susanne Zock-Emmenthal, Martin Lützelberger, Susann Plath, Henning Schmidt, Angel Guerra-Moreno, Luca Cozzuto, José Ayté, Norbert F. Käufer

**Affiliations:** 1 Institute of Genetics, Technische Universität Braunschweig, Braunschweig, Germany; 2 Department of Physiology and Pathophysiology, Faculty of Medicine, University of Manitoba, Winnipeg, Canada; 3 Oxidative Stress and Cell Cycle Group, Universitat Pompeu Fabra, Barcelona, Spain; 4 CRG Bioinformatics Core, Centre de Regulació Genòmica (CRG), and Universitat Pompeu Fabra, Barcelona, Spain; The University of North Carolina at Chapel Hill, UNITED STATES

## Abstract

The genome of the fission yeast *Schizosaccharomyces pombe* encodes 17 kinases that are essential for cell growth. These include the cell-cycle regulator Cdc2, as well as several kinases that coordinate cell growth, polarity, and morphogenesis during the cell cycle. In this study, we further characterized another of these essential kinases, Prp4, and showed that the splicing of many introns is dependent on Prp4 kinase activity. For detailed characterization, we chose the genes *res1* and *ppk8*, each of which contains one intron of typical size and position. Splicing of the *res1* intron was dependent on Prp4 kinase activity, whereas splicing of the *ppk8* intron was not. Extensive mutational analyses of the 5’ splice site of both genes revealed that proper transient interaction with the 5’ end of snRNA U1 governs the dependence of splicing on Prp4 kinase activity. Proper transient interaction between the branch sequence and snRNA U2 was also important. Therefore, the Prp4 kinase is required for recognition and efficient splicing of introns displaying weak exon1/5’ splice sites and weak branch sequences.

## Author Summary

Prp4 is an essential protein kinase that is involved in the splicing of some introns. Using a conditional mutant of Prp4, we showed that a subset of genes, including several cell cycle–regulatory genes, are dependent on Prp4 for splicing. Furthermore, we could convert genes between Prp4-dependent and -independent states by introducing single-nucleotide mutations in the exon1/5’ splice sites and branch sequence of introns. This work shows that Prp4 activity is required for splicing surveillance in a subset of mRNAs.

## Introduction

Introns are removed from pre-mRNAs by spliceosomes, which are highly dynamic macromolecular complexes consisting of five small nuclear RNAs (snRNAs; U1, U2, U5, and U4/U6) associated with specific proteins in subcomplexes called snRNPs. *In vitro*, spliceosomal subcomplexes assemble on pre-mRNAs in a time-dependent manner. The intron to be removed is defined during the formation of spliceosomal complex B, which contains the base-paired U4/U6 snRNP as well as the other three snRNPs. ATP-dependent helicases control the specific rearrangement of the B complex so that catalysis of the transesterification reactions can occur in spliceosomal complex C, formed by the departure of snRNPs U1 and U4 and rearrangement of snRNPs U2, U6, and U5, culminating in the splicing reaction [1–3]. Although pre-mRNA splicing is an important part of regulated gene expression, little is known about the assembly and activation of spliceosomes *in vivo*. Introns are presumably recognized and removed by the spliceosome during or shortly after transcription, suggesting that parts of the spliceosomal complex must be recruited to transcribed chromatin areas, installed at introns, and then activated for catalysis. Several lines of evidence suggest that the 5’ splice site (SS) is defined by direct interactions with snRNA U1, and that definition of the 3’ SS also involves the interaction of the branch sequence (bs) with snRNA U2 [4,5].

In the fission yeast *Schizosaccharomyces pombe*, approximately 45% of genes contain at least one intron, with some genes containing as many as 15. Compared with the introns in budding yeast *Saccharomyces cerevisiae*, fission yeast introns are relatively small: the average intron sizes in *S*. *cerevisiae* and *S*. *pombe* are 256 nt and 83 nt, respectively. In *S*. *cerevisiae*, only 5% of genes contain introns, and the 5’ SS and bs at the 3’ region of the intron are highly conserved; in contrast, in *S*. *pombe*, the 5’ SS and bs are as variable as the corresponding sequences in mammals [6–8]. Regulated alternative splicing has not been observed in mitotically active fission yeast cells [9]; however, certain genes appear to be regulated by splicing during sexual differentiation. The 80 nt intron in the pre-mRNA encoding the meiotic cyclin Rem1, for example, is spliced after the initiation of meiosis when the gene is transcribed by the Forkhead family transcription factor Mei4 [10,11].

We identified and characterized the Prp4 kinase, which phosphorylates the spliceosomal protein Prp1 (scPrp6/hsU5-102K) *in vitro* and *in vivo*. Prp4 also phosphorylates Srp2, one of the two typical SR (serine/arginine-rich) protein family members present in fungi [12,13]. Prp1 contains 16 direct C-terminal tetratricopeptide repeats (TPRs), preceded by an N-terminal domain of approximately 30 kDa that contains no known motifs. The C-terminal region containing the TPRs is highly conserved across a wide range of organisms, whereas the N-terminal region is not [13]. Prp4 phosphorylates Prp1 at sites in the N-terminal region [14,15]. In fission yeast, the structural integrity of the N-terminal domain is essential for pre-mRNA splicing. Short deletions throughout the N-terminus of Prp1 do not prevent spliceosome assembly but lead to the formation of stalled precatalytic spliceosomes that contain unspliced pre-mRNA and the U1, U2, U5, and U4/U6 snRNAs, indicating that the N-terminus of Prp1 is involved in early spliceosome activation [14]. In mammals, the Prp4 ortholog Prp4K phosphorylates equivalent sites in the N-terminal region of Prp1, and also phosphorylates SR proteins [13,16,17].

SR proteins contain one or two N-terminal RNA recognition motifs (RRMs) and a C-terminal RS domain enriched in arginine–serine dipeptides [18]. In general, SR proteins control a network of RNA processing events, including the regulation of SS selection [19]. In mammals, they can act as splicing enhancer or silencer depending on their position of binding [20]. It is known that they play an important role not only in alternative splicing but also in constitutive splicing [21,22]. In case of constitutive splicing they take part in intron recognition at the exon1/5´ SS by interacting with hsU1-70K (spUsp101) as well as at the 3´ SS by interacting with hsU2AF1 (spUaf2) [23–26]. In *S*. *pombe* there are two SR proteins known, Srp1 (hsSRSF2) and Srp2 (hsSRSF4/5/6) [27,28]. They were found as single proteins but also as a complex depending on their phosphorylation states [29]. Srp2 is known to be phosphorylated by Prp4 kinase while Srp1 is not [13]. However, it was shown that overexpression of Srp1 can suppress the splicing phenotype of the mutant allele *prp4-73*^*ts*^ [30]. The temperature-sensitive allele *prp4*^*ts*^ caused at the restrictive temperature of 36°C a cell-cycle arrest in the G1 and G2 phases. This phenotype was also observed at the permissive temperature of 25°C when the mutant allele *prp4-73*^*ts*^ was expressed from a multicopy plasmid [15].

Cell-cycle arrest in the G1 and G2 phase has been observed primarily in mutants of genes involved in cell-cycle regulation. Therefore, in our attempt to elucidate the mechanism underlying this phenotype, we first examined the splicing of cell-cycle regulatory genes that contain introns, e.g., *cdc2*, *res1*, and *res2*. We performed these experiments in cells expressing the analogue-sensitive (as) mutant *prp4-as2*, which encodes a kinase that can be chemically inhibited. This analysis revealed two classes of introns in fission yeast: those whose splicing is dependent on Prp4 kinase activity (Prp4-dependent) and those whose splicing is independent of Prp4 kinase activity (Prp4-independent). This finding was confirmed and extended by a genome-wide search for Prp4-dependent and -independent introns, which demonstrated that both intron classes are sometimes present within the same gene. For detailed characterization, we focused on two genes, *res1* and *ppk8*; the single *res1* intron is Prp4-dependent, whereas the single *ppk8* intron is Prp4-independent. These two intron classes are affected by mutations in the exon1/5’ SS region or the bs of the same intron. The potential interactions between the exon1/5’ SS region and snRNP U1, and between the bs and snRNP U2, determine the Prp4 dependency of the intron. Taking into consideration the results of this and previous studies, we propose that phosphorylation of different substrates by Prp4 kinase helps the spliceosome to recognize and splice efficiently introns with weak SSs that differ from the consensus sequence.

## Results

### Chemical inhibition of Prp4 kinase causes transient arrest in the G1 and G2 phases of the cell cycle

**Fig 1:**
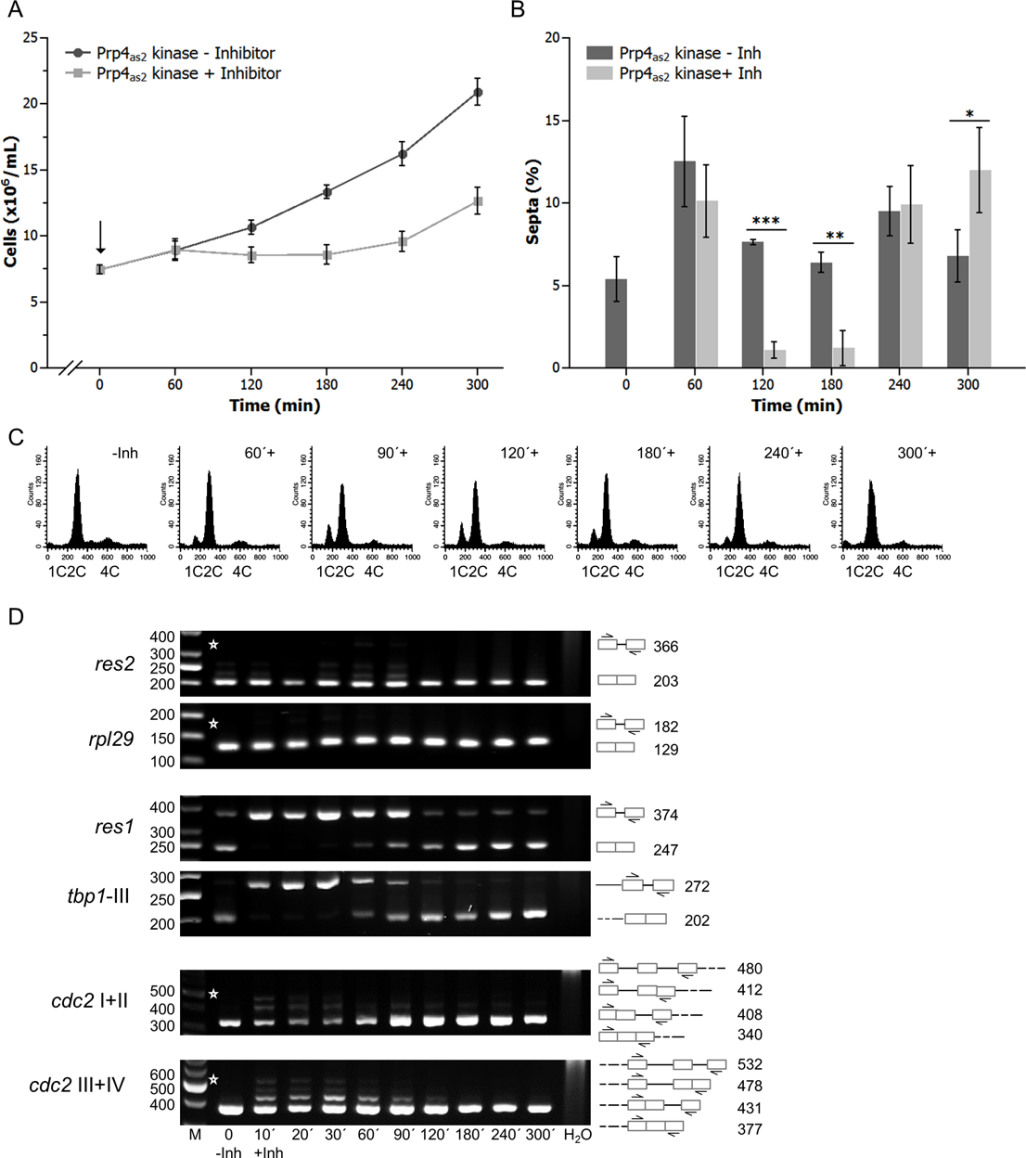
Prp4_as2_ kinase and its inhibition with 1NM-PP1 in fission yeast. (A) A strain with the genotype *h*^*−s*^ *prp4-as2* was grown at 30°C to early log-phase. The inhibitor 1NM-PP1 was then added to the culture medium (0 hours, arrow, **↓**) at a final concentration of 10 μM. Growth of the culture was monitored by counting the number of cells/mL (squares) relative to a culture growing in the absence of inhibitor (circles). The error bars indicate standard deviation. (B) Percentage of septated cells during growth in the absence (-Inh) and presence (+Inh) of inhibitor. Bars show the mean value of three independent repetitions (n = 3) and error bars indicate the standard deviation. A two-tailed t-test was performed to check whether the number of septated cells differs significantly without and with inhibition of the kinase (* = p < 0.05; ** = p < 0.01; *** = p < 0.001). (C) DNA content analysis in C of *prp4-as2* cells immediately before (-Inh) and at the indicated times after the addition of 1NM-PP1 (+). (D) RT-PCR analyses of RNA prepared at the indicated times after the addition of inhibitor (+Inh). RNA was also extracted from cells grown in the absence of inhibitor (-Inh). Specific primers were used to detect *res2*, *rpl29*, *res1*, *tbp1*-III, *cdc2* I+II and *cdc2* III+IV RNAs. Roman numerals indicate the intron numbers contained within the amplicons. The numbers on the right side of the image represent the sizes of the RT-PCR fragments (bp). Asterisks indicate the expected positions of fragments if the introns between the indicated primer pairs are not spliced out. H_2_O, negative control without template. The numbers on the left side of the image represent the sizes of the DNA fragments (bp). M, DNA size marker.

For these experiments, we constructed a conditional analogue-sensitive allele, *prp4-as2*, which allows reversible inhibition of Prp4 kinase using an ATP analogue. To inhibit Prp4, 10 μM of 1NM-PP1 was added to growing cultures of cells expressing the *prp4-as2* allele. Addition of inhibitor caused growth arrest and a concomitant decrease in the number of septated cells (Fig 1A and 1B). Cells arrested in the G1 and G2 phases of the cell cycle, as observed for the *prp4*^*ts*^ strain. Indeed, fluorescence-activated cell sorting (FACS) analysis revealed cells with both 1C and 2C DNA content after 60 min inhibition of Prp4 (Fig 1C). This growth arrest was transient, and cells resumed growing after approximately 180 min, as indicated by the disappearance of the 1C peak and the reappearance of septated cells after 240 min (Fig 1).

The transient arrest in the G1 and G2 phases of the cell cycle led us to hypothesize that the genes whose splicing was affected by Prp4 inhibition were directly or indirectly involved in mitotic cell-cycle progression. For example, the gene encoding the cell-cycle regulator Cdc2 contains four introns that interrupt its open reading frame. Oscillating Cdc2 kinase activity levels regulate cell-cycle progression in G1 and G2 [31]. The decision point at which cells either make the transition from G1 phase to DNA synthesis or exit the cell cycle for conjugation is known as START. Cdc2 kinase and components of the Mlu1-binding factor (MBF) are involved in control of START by regulating the expression of genes required for DNA replication. The multimeric MBF complex consists of Cdc10, Res1, and Res2 [32,33]. The *cdc10* gene is intronless, whereas the *res1* and *res2* genes each contain a single intron (of 127 nt and 164 nt, respectively) located in the 5’ region of the open reading frame [34,35].

Thus, we focused on the splicing of intron-containing genes that regulate the transition from G1 phase to DNA synthesis. Semiquantitative reverse transcription polymerase chain reaction (RT-PCR) analyses were performed to measure the mRNA and pre-mRNA levels of *res1*, *res2*, *cdc2*, and two control genes, *rpl29* and *tbp1* (Fig 1D). *rpl29* contains one intron and encodes large ribosomal subunit protein 29; *tbp1* contains three introns and encodes the TATA-binding protein [36]. The highly efficient splicing of *res2* and *rpl29* were barely affected by inhibition of Prp4 kinase. By contrast, for *res1* and *tbp1*, unspliced pre-mRNAs accumulated and almost no mRNA was detected, as little as 10 min after the addition of inhibitor. This strong inhibition of splicing was transient, and mature spliced mRNA transcripts of both genes were observed again after 60 min. After 180 min, spliced mRNA levels were similar to those observed in the absence of inhibition (Fig 1D). Splicing of all four introns of the *cdc2* transcript was only slightly affected by inhibition of Prp4 kinase, and mature *cdc2* mRNA was detected at all time points. Remarkably, the splicing pattern of *res1* and *tbp1* remained basically the same throughout the time course (Fig 1D).

Collectively, these results indicated that the introns of the five genes we investigated could be categorized into two classes: Prp4-dependent and Prp4-independent. Subsequent experiments showed that the *res1* intron was primarily responsible for the cell-cycle arrest in G1 following Prp4 inhibition: replacing the wild-type *res1* gene with an intronless copy (*res1Δintron*) led to a similar growth delay, but the cells now primarily arrested in G2 phase (S1 Fig).

### Prp4-dependent and -independent introns throughout the genome

**Fig 2:**
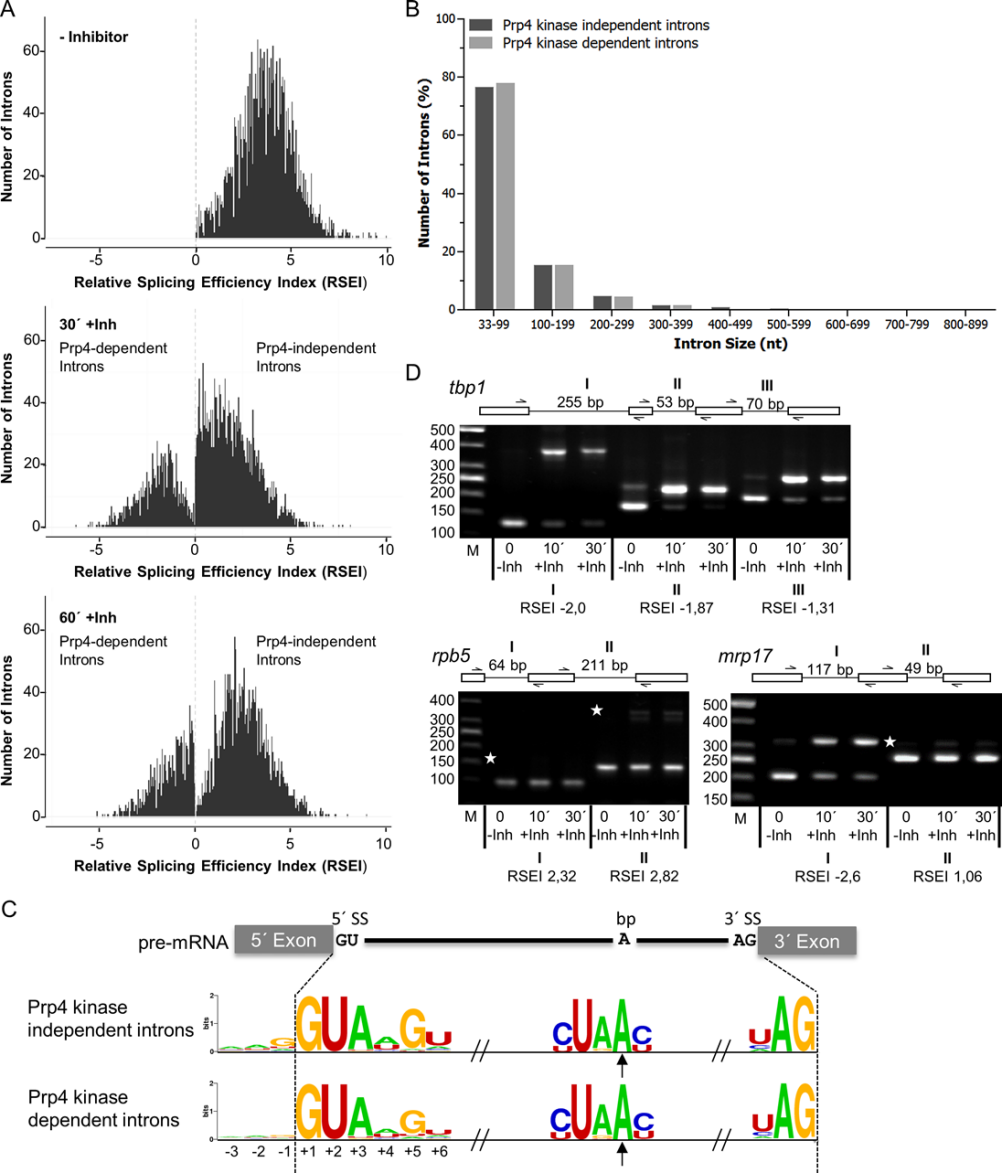
Whole-genome splicing profile of a fission yeast strain expressing Prp4_as2_ kinase. (A) The frequency histograms assign the number of introns found for each calculated Relative Splicing Efficiency Index (RSEI) in the absence (- Inhibitor) of 1NM-PP1 or after 30 and 60 min in the presence of inhibitor. The bin size is 0.05. Bars with negative RSEI values display Prp4-dependent introns (1008 introns) while bars with positive RSEI values represent Prp4-independent introns (2557 introns). The dashed line marks the 0 value. (B) Similar size distributions of Prp4-independent and -dependent introns. (C) A hypothetical example of a fission yeast pre-mRNA. Consensus sequences of the 5’ SS, branch sequence with branch point A (bp, arrow), and 3’ SS are shown. These consensus sequences do not differ between Prp4-dependent and–independent introns. The sequence logos were generated using WebLogo [74]. (D) Splicing of the introns of *rpb5*, *tbp1*, and *mrp17* monitored by RT-PCR using specific primers, as indicated in the schemes above the images, in the absence of inhibitor (-Inh) or after 10 and 30 minutes in the presence of inhibitor (+Inh). Asterisks indicate the expected position of fragments if the introns are not spliced out. RSEI below the images was obtained from cells collected after 30 min in the presence of inhibitor. Roman numerals indicate the 5’→3’ order of introns. The numbers on the left side of the image represent the sizes of the DNA fragments (bp). M, DNA size marker.

To further examine these two classes of introns, we performed a genome-wide search for additional Prp4-dependent and–independent introns. RNA prepared from the *prp4-as2* strain grown in the presence (30 min and 60 min exposure, +Inh) or absence (-Inh) of the 1NM-PP1 inhibitor were subjected to RNA sequencing (RNA-seq), and the resultant sequence reads were aligned to the spliced or unspliced fission yeast genome reference sequence. To examine global changes in splicing efficiency, the Relative Splicing Efficiency Index (RSEI) of annotated fission yeast introns was calculated for the untreated and treated datasets. Fission yeast introns were divided into two classes. The first class contained the 72% of all introns with a positive RSEI in the presence and absence of 1NM-PP1, indicating that splicing of these introns was Prp4-independent. This class included *res2*, *rpl29*, *and cdc2* (Fig 2A). The second class contained the 28% of introns for which RSEI was positive in untreated cells but negative in cells treated with 1NM-PP1, indicating that splicing of these introns was Prp4-dependent. This class included *res1* and *tbp1* (Fig 2A). Notably, we were unable to identify any gross sequence features that differentiated these two classes of introns. For example, there were no significant differences in intron size or obvious additional sequence motifs that were specific to either class (Fig 2B). Comparison of the SS sequences of Prp4-independent and -dependent introns revealed only slight differences between the two classes especially at position -1 in the exon 1 and positions +4 to +6 in the 5´ SS. The Prp4-dependent introns differed more frequently at these positions from the consensus sequence compared to Prp4-independent ones (Fig 2C). Moreover, in genes containing several introns, not all introns necessarily behaved in the same way upon inhibition of Prp4. This different behaviour of introns within one gene was also observed for temperature-sensitive alleles of other splicing factors [37,38]. As shown in Fig 2D, the *mrp17* gene, encoding mitochondrial ribosomal subunit Mrp17, has one Prp4-dependent and one Prp4-independent intron; by contrast, *rpb5*, encoding a DNA-directed RNA polymerase subunit, contains two Prp4-independent introns, and *tbp1* contains three Prp4-dependent introns.

### Prp4-dependent and -independent introns can be interconverted by mutating the exon1/5’ SS region

**Fig 3:**
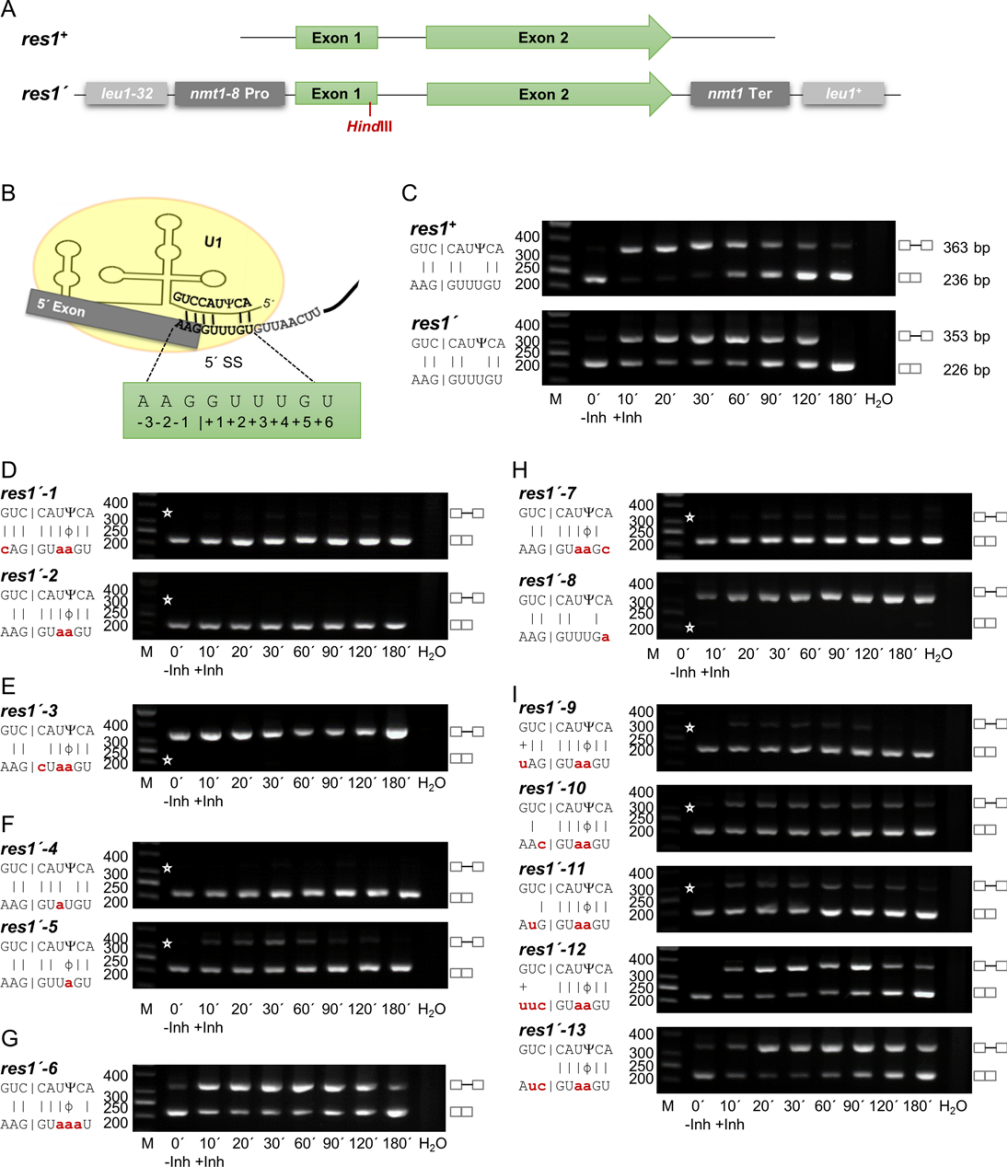
The Prp4 kinase dependence of the *res1* intron can be changed by mutations in the exon1/5’ splice site. (A) Schematic representation of the *res1*^*+*^ and *res1’* genes. The *res1’* gene was integrated by homologous recombination into the *leu1* locus. Because Res1 is essential for growth, all strains containing the *res1’* gene also contain *res1*^*+*^. (B) Proposed base-pairing between the *res1*^*+*^ exon1/5’ SS region and snRNA U1. Ψ indicates the pseudouridine 3 nucleotides from the 5’ end of snRNA U1. Numbering of the exon1/5’ SS region is indicated. (C–I) RT-PCR analysis in the absence (-Inh) and presence (+Inh) of inhibitor at the indicated times. H_2_O, negative control without template. The scheme on the left side of the image shows the details of the interactions between the exon1/5’ SS region and snRNA U1. Small letters indicate the mutations in the *res1’* exon1/5’ SS; the corresponding alleles were named as indicated. |, Watson-Crick base-pairing; +, wobble base-pairing G-U; Ψ, Pseudouridine; ϕ, wobble base-pairing Ψ-A. Asterisks indicate the expected position of fragments if the introns are or are not spliced out. The numbers on the left side of the image represent the sizes of the DNA fragments (bp). M, DNA size marker. C compares the endogenous *res1*^*+*^ with the integrated *res1’*.

**Fig 4:**
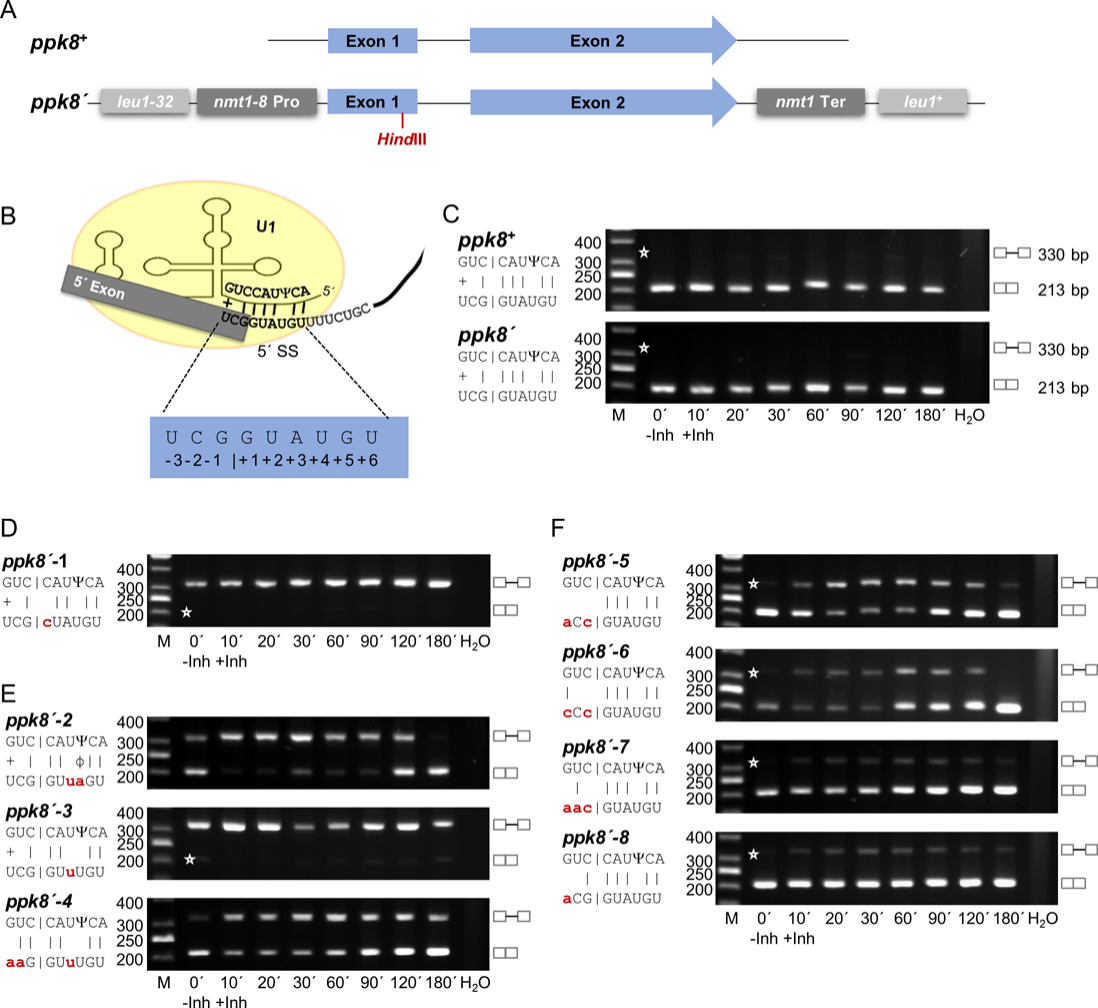
The Prp4 kinase independence of the *ppk8* intron can be changed by mutations in the exon1/5’ splice site. (A) Schematic representation of the *ppk8*^*+*^ and *ppk8’* genes. The *ppk8’* gene was integrated by homologous recombination into the *leu1* locus. All strains containing a *ppk8’* gene also contain *ppk8*^*+*^. (B) Proposed base-pairing between the *ppk8*^*+*^ exon1/5’ SS region and snRNA U1. Ψ indicates the pseudouridine 3 nucleotides from the 5’ end of snRNA U1. Numbering of the exon1/5’ SS region is indicated. (C–F) RT-PCR analysis in the absence (-Inh) and presence (+Inh) of inhibitor at the times indicated in minutes. H_2_O, negative control without template. The scheme on the left side of the image shows the details of the interactions between exon1/5’ SS and snRNA U1. Small letters indicate the mutations in *ppk8’* exon1/5’ SS; the corresponding alleles were named as indicated. |, Watson-Crick base-pairing; +, wobble base-pairing G-U; Ψ, Pseudouridine; ϕ, wobble base-pairing Ψ-A. Asterisks indicate the expected position of fragments if the introns are or are not spliced out. The numbers on the left side of the image represent the sizes of the DNA fragments (bp). M, DNA size marker. C compares endogenous *ppk8*^*+*^ with integrated *ppk8’*.

We wished to characterize the changes in splicing efficiency when the exon1/5’ SS region and bs of these two intron types were mutated. For these experiments, we selected two genes, each containing one intron of similar size and structure, but with RSEI of opposite sign following inhibition of Prp4 kinase. The Prp4-dependent gene was *res1*, which contains a 127 nt intron, has an RSEI of -1.36, and is essential for mitotic growth (Fig 3A). The Prp4-independent gene was *ppk8*, which contains a 117 nt intron and has an RSEI of +1.89 (Fig 4A). This gene is non-essential for mitotic growth; it encodes a putative serine/threonine kinase potentially involved in signal transduction [39]. Because we wanted to compare the pre-mRNA splicing of these functionally very different genes following mutation, we constructed two reporter genes, called *res1’* and *ppk8’*, respectively. Both reporter genes are driven by the *nmt1-8* promoter and contain a frameshift mutation early in exon 1, a *Hin*dIII restriction site upstream of the 5’ SS, and the *nmt1* termination region for 3’ end processing. These manipulated genes were introduced into the *leu1* locus by homologous recombination (Figs 3A and 4A), and semiquantitative RT-PCR analyses were performed using the appropriate primers. As shown in Fig 3B–3C, the *res1’* intron at the *leu1* locus was spliced in a Prp4-dependent manner, like endogenous *res1*^*+*^, whereas the intron of *ppk8’* was Prp4-independent like endogenous *ppk8*^*+*^ (Fig 4B–4C). This is consistent with the notion that the Prp4 dependency of a gene is not governed by its genomic context: neither chromosomal location nor the identity of the promoter and 3’ termination region determined whether an intron was spliced in a Prp4-dependent manner. Therefore, the differences in Prp4 dependency must be due to subtle differences in or around the intronic sequences.

Base-pairing interactions between pre-mRNA and snRNA U1 play a role in establishing and determining the 5’ SS [40–42]. This recognition process is more complicated in mammals than in yeast, because alternative pre-mRNA splicing requires selection of one out of several possible 5’ SSs [43]. In fungi, however, and particularly in fission yeast, there is little or no alternative splicing [9]. In fission yeast, it has been suggested that nine nucleotides from the 5’ end of the U1 snRNA interact with the pre-mRNA, base-pairing with six nucleotides of the 5’ SS and three nucleotides of exon 1, to determine the 5’ SS [44]. In addition, the 5’ end of snRNA U1 in fission yeast becomes pseudouridinylated (Ψ). This mechanism is also conserved in eucaryotes. However, in mammalian U1 snRNA two adjacent nucleotides in this region are pseudouridinylated, whereas in fission yeast, only nucleotide number 3 from the 5’ end of snRNA U1 is pseudouridinylated. This nucleotide in the U1 snRNA interacts with the 5’ SS nucleotide +4 of the pre-mRNA (Figs 3B and 4B). In general, pseudouridinylated nucleotides base-pair with A, C, G and U in an A-form RNA duplex, but the highest thermal stability can be found between A-Ψ and G-Ψ [45–48].

To analyse if an increased base-pairing potential leads to a Prp4-independent intron and vice versa, several mutations were introduced into the exon1/5´ SS of the reporter genes *res1´* and *ppk8´* (Figs 3 and 4). We mutated positions -3, +3 and +4 (Fig 3D, *res1´-1)* or only positions +3 and +4 (Fig 3D, *res1´-*2) of the *res1’* intron. These changes, which increased the potential interactions between U1 snRNA and the pre-mRNA by at least four hydrogen bonds, converted the *res1’* intron into a Prp4-independent intron. A time-course RT-PCR experiment revealed that these mutations allowed efficient splicing in the presence (+Inh) or absence (-Inh) of inhibitor (Fig 3D). As a control, we also mutagenized position +1 or +2 of the *res1’* intron to a C or A, respectively; the resultant mutants were not recognized efficiently as introns independent of Prp4 activity (Fig 3E and S2 Fig, *res1´-14*). At the time that introns were discovered, it was shown that the GU at the 5’ end is necessary for recognition of an intron [49]. Therefore, this control experiment confirms our interpretation that the transient interaction of the exon1/5’ SS region with snRNA U1, including nine nucleotides from the 5’ end of U1, allows efficient splicing in the absence of Prp4 kinase activity (compare Fig 3D, *res1’-1* and *res1’-2* with Fig 3E). To test whether only one additional interaction at position +3 or +4 is already sufficient for Prp4 independency, both mutants were constructed and resulted in Prp4-independent introns. While an interaction at position +3 lead to an efficiently spliced intron at all time points, an interaction at position +4 caused a slightly decreased splicing efficiency after inhibition (Fig 3F). For all further experiments the Prp4-independent *res1´-2* exon1/5´ SS was used to analyse which additional mutations lead to Prp4 dependency again. When these continuous interactions were interrupted by mutating position +5 in the intron from a G to an A, the intron became Prp4-dependent once again (Fig 3G). All mutations at position +5 that did not allow Watson–Crick hydrogen bonding with a C at position 2 of snRNA U1 caused the *res1’* intron to be Prp4-dependent (Fig 3G and S2 Fig, *res1’-15* and *res1’-16*); therefore, these experiments also prove that proper interaction between U1 and the exon1/5’ SS region is established by formation of hydrogen bonds between the two opposing bases. By contrast, the Prp4 independence of this intron was unaffected by all mutations at position +6 (the last 5’ SS nucleotide) that did not allow hydrogen bonding with the A at position 1 of the U1 snRNA (Fig 3H, *res1´-7* and S2 Fig, *res1’-17* and *res1’-18*). But if there is no interaction at positions +3, +4 and +6 the former Prp4-dependent intron (Fig 3C, *res1´*) is not recognized anymore even in presence of Prp4 kinase activity (Fig 3H, *res1´-8*). Thus, increasing the base-pairing potential between U1 snRNA and pre-mRNA within the 5´ SS results in Prp4-independently spliced introns and vice versa. Similar, rules seemed to apply for mutations at positions -1, -2 and -3 of exon 1 of the *res1´* gene (Fig 3I). If only position -1 or position -2 can form a Watson–Crick hydrogen bond, therefore creating a weaker interaction in the exon 1 compared to *res1´-2*, splicing efficiency decreased after inhibition of Prp4, but the intron was still spliced independently (Fig 3I, *res1’-10* and *res1’-11*). However, completely absent or very weak hydrogen bonding with the nucleotide at position -3 of exon 1 caused the intron to be Prp4-dependent (Fig 3I, *res1’-12* and *res1’-13*).

Like *ppk8*, the intron of the *ppk8’* gene integrated into the *leu1* locus was also Prp4-independent (Fig 4A–4C). First, the 5’ SS nucleotide +1 G was mutated to a C, the intron was no longer recognized regardless of Prp4 kinase activity, as demonstrated by the exclusive presence of pre-mRNA and absence of mature mRNA (Fig 4D). Then position +3 was mutated from an A to a U, preventing Watson–Crick hydrogen bonding with the snRNA U1, and position +4 from a U to an A, creating an A-Ψ interaction (Fig 4E, *ppk8’-2*). This shows that changing the position of two hydrogen bonds of the 5´ SS converted a Prp4-independent intron into a Prp4-dependent one (compare Fig 4C, *ppk8´* with 4E, *ppk8´-2*). When the interactions at both positions, +3 and +4, were absent, the resultant intron was no longer recognized (Fig 4E, *ppk8’-3*). However, changing the interactions within the exon 1 creating a *ppk8’* gene containing the exon1/5’ SS sequence of the *res1*^*+*^ gene was Prp4-dependent (Fig 4F, *ppk8’-4*).

Next, we introduced mutations at the end of exon 1 to determine the effect of the pairing of these nucleotides on Prp4 dependency. If the nucleotides at positions -1, -2, and -3 of exon 1 in *ppk8’* were unable to form hydrogen bonds with the appropriate positions in snRNA U1, the intron was spliced in a Prp4-dependent manner (Fig 4F, *ppk8’-5*). The same rule applied if hydrogen bonding only occurred at position -3 (Fig 4F, *ppk8’-6*). However, if a potential hydrogen bond could be formed with the nucleotide at position -1 or -2, stabilizing the interactions between exon 1 and snRNA U1, the intron was spliced efficiently in a Prp4-independent manner (Fig 4F, *ppk8’-7* and *ppk8´-8*). Taken together, these results clearly demonstrate that the number and position of the potential hydrogen bonds between the nucleotides of the exon1/5´ SS region of an intron and the 5’ end of the U1 snRNA is one reason whether an intron is spliced in a Prp4-independent or -dependent manner.

### A Prp4-dependent intron can be created by introducing mutations in the branch sequence

**Fig 5:**
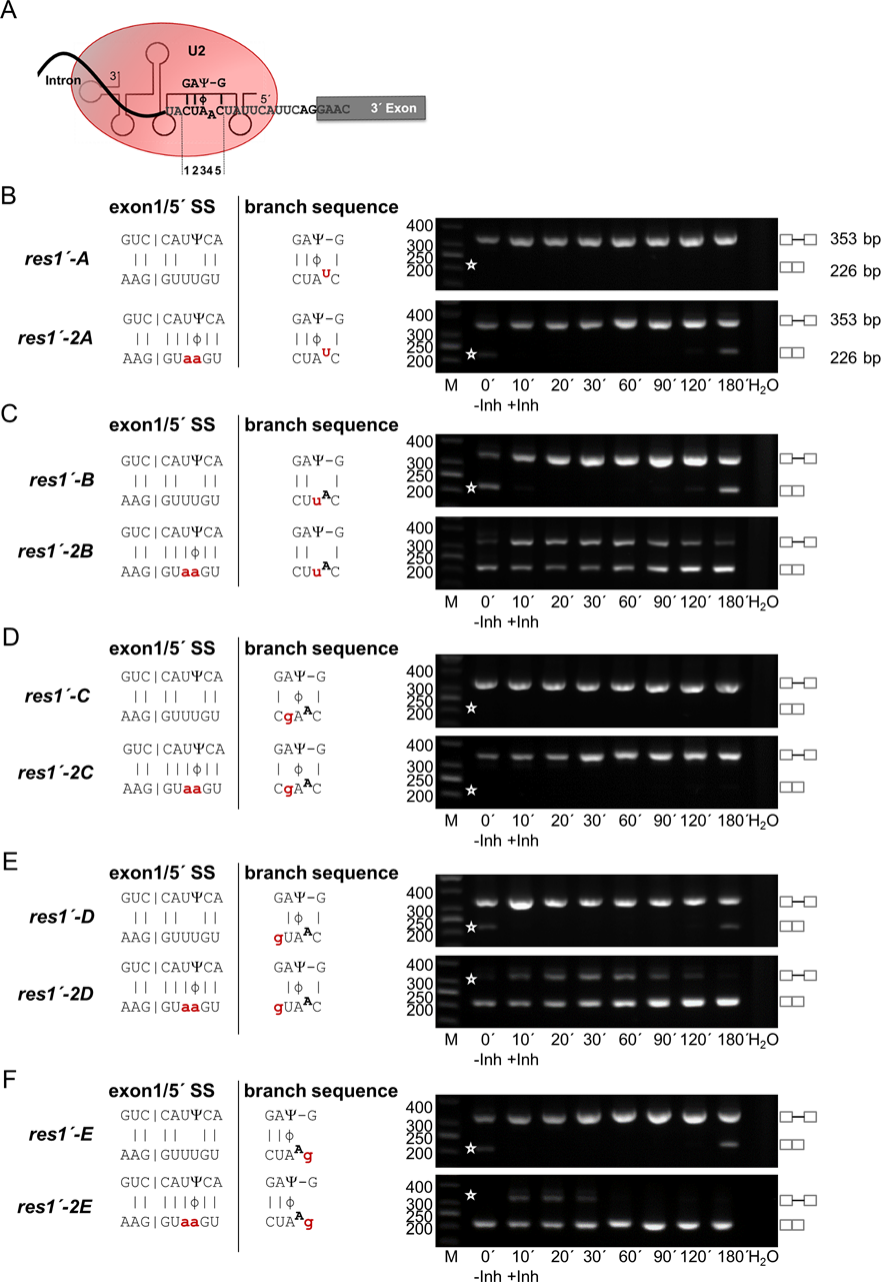
Point mutation in the third position of the branch sequence converts a Prp4 kinase-independent intron into a kinase-dependent intron. (A) Proposed base-pairing between the *res1* intron branch sequence CUAAC and snRNA U2. Ψ indicates the pseudouridine 39 nucleotides from the 5’ end of snRNA U2, which is suggested to base-pair with the A at position 3 of the branch sequence. (B) *res1’-A* and *res1’-2A*: RT-PCR analysis in the absence (-Inh) and presence (+Inh) of inhibitor at the indicated times. (C) *res1’-B* and *res1’-2B*. (D) *res1’-C* and *res1’-2C*. (E) *res1’-D* and *res1’-2D* (F) *res1’-E* and *res1’-2E*. H_2_O, negative control without template. The scheme on the left side of the images show the details of the interactions between exon1/5’ SS and snRNA U1 and between the branch sequence and snRNA U2. Small letters indicate the mutations in exon1/5’ SS and the branch sequence; the corresponding alleles were named as indicated. |, Watson-Crick base-pairing; Ψ, Pseudouridine; ϕ, wobble base-pairing Ψ-A. Asterisks indicate the expected position of fragments if the introns are or are not spliced out. The numbers on the left side of the image represent the sizes of the DNA fragments (bp). M, DNA size marker.

As discussed above, we did not find any obvious differences in the bs consensus between +RSEI and–RSEI introns (Fig 2C). The consensus branch sequence of *S*. *pombe* is 1.C/U 2.U 3.A/G/U/C 4.A 5.C/U; the most frequent bs sequences are CUAAC (42%) and CUAAU (23%) [39,50]. The branch point A is the fourth nucleotide in this sequence [51–53]. The 5 nt degenerate bs of fission yeast is similar to that of mammals [6,8]. Recognition of the bs via base-pairing of snRNA U2 is also conserved, and the nucleotide at position 3 in the bs of *S*. *pombe* is opposite a pseudouridine in snRNA U2 [45,47,54]. The bs of the *res1* intron has the most common sequence (CUAAC), and the pseudouridine in snRNA U2 is at position 39 from the 5’ end (Fig 5A). To study the influence of mutations in the bs on Prp4 dependency, we used *res1´* transcripts with exon1/5´ SS regions AAG/GUAAGU (Prp4-independent) and AAG/GUUUGU (Prp4-dependent), respectively. We combined mutations in the 5´ SS with mutations in the bs. As expected, mutation of the branch point in position 4 (from A to U) combined with the Prp4-dependent 5’ SS prevented recognition of the intron, regardless of the presence or absence of inhibitor (Fig 5B, *res1’-A*). The reporter gene carrying the Prp4-independent 5’ SS sequence was spliced extremely inefficient, and it was further inhibited by inactivation of Prp4 (+Inh); consequently, only a small amount of mRNA was detected at the end of the time course (Fig 5B, *res1’-2A*).

The third nucleotide in the bs, which is degenerate for A, G and U, is supposed to interact with the pseudouridine at position 39 in snRNA U2. When we mutated this nucleotide from A to U, we converted the Prp4-independent intron into a Prp4-dependent one (compare Fig 5C, *res1´-2B* with Fig 3D, *res1’-2*). In contrast, the *res1’-B* gene, which already contains the Prp4-dependent exon1/5’ SS region, was inefficiently spliced even in the absence of Prp4 inhibitor. After addition of kinase inhibitor pre-mRNA accumulated completely (Fig 5C, *res1’-B*). These findings are in accordance with the notion that mutations in the third position of the bs lead to a Prp4-dependent intron when there is no Ψ-A interaction. In addition, if the intron was already Prp4 kinase-dependent because of a weak interaction between the exon1/5’ SS region and snRNA U1, the mutation in the bs intensifies the effect (Fig 5C, *res1´-B*).

The second nucleotide (U) in the bs is 100% conserved in all *S*. *pombe* introns. When we changed this U to a G, the intron was no longer recognized, neither without nor with Prp4 kinase inhibitor (Fig 5D). Furthermore, mutations in the first or the last nucleotide of the bs combined with the Prp4-dependent exon1/5´ SS lead to an extremely inefficient splicing event which was further intensified after inhibition of the kinase (Fig 5E, *res1’-D* and Fig 5F, *res1’-E*). However, when combined with the Prp4-independent exon1/5’ SS these mutations were spliced independently, but after inhibition of the kinase the splicing efficiency decrease slightly (Fig 5E, *res1’-2D* and Fig 5F, *res1’-2E*). To summarize the influence of mutations within the bs it is obvious that in case of a weak exon1/5´ SS the negative effect on intron recognition is intensified which leads mostly to intron retention. In contrast mutations in the bs combined with a strong exon1/5´ SS show different results depending on the position of the mutations. Except position 2 all others show an improvement of intron recognition as long as Prp4 kinase is active.

## Discussion

The 5’ SS and the bs of the introns in *S*. *cerevisiae* are highly conserved (GUAUGU and UACUAAC, respectively, in almost all introns) [53,55,56], whereas the corresponding sequences in *S*. *pombe* are much more degenerate; GUAAGU and GUAUGU are the most frequent 5’ SSs (29% and 21% of all introns, respectively), and CUAAC is the most frequent bs (42%) [39]. In this context we have shown that Prp4 kinase is one of the major components to facilitate proper recognition of introns with weak SSs. This kinase is involved in the process that helps to sense and influence proper base-pairing between the exon 1/5’ SS region and snRNA U1 and between the bs and snRNA U2; in this background, proper base-pairing refers to an interaction that accurately determines the 5’ and 3’ SS, leading to an efficient splicing event.

In this study we have shown by mutating the exon1/5’ SS and the bs of reporter genes that a Prp4-dependent intron can be changed into an–independent one and vice versa (Fig 3C and 3D and Fig 4C and 4E). The experimental set up also demonstrates that the information for Prp4 dependency resides in the region of the SSs of an intron and has been confirmed by inserting introns with weak and strong SSs into an intronless gene (S3 Fig). Regarding Prp4 dependency the positions for proper base-pairing between the exon1/5’ SS region and snRNA U1 play different roles. For example, positions +1 and +2 are invariable as it was known before [49]. Mutations of these positions lead to accumulation of unspliced pre-mRNA in the presence and absence of Prp4 kinase activity (Figs 3E, 4D and S2, *res1´-14*). This is different, if one considers position +3 and +4. Position +4 in the 5’ SS is the most degenerate, and can be occupied by any of the four nucleotides (Fig 2C). This position has been suggested to appear opposite a pseudouridinylated nucleotide of snRNA U1 (Fig 3B). Pseudouridine can base-pair with all nucleotides, although the thermodynamic parameters of these wobble base-pair interactions are different from those of Watson–Crick interactions [46,48,54]. If there is only one Watson-Crick interaction present at position -1 in exon 1 and the base-pairing at position +4 in the 5´ SS is interrupted, the intron is spliced Prp4-independently (Fig 4C). In contrast, an interruption at position +3 leads to a Prp4-dependent intron (Fig 4E, *ppk8´-2*). However, if there are two Watson-Crick interactions in exon 1, at positions -1 and -2, it does not matter which position, +3 or +4, is mutated (Fig 3F). The intron is spliced Prp4-independently. These results show that a stable interaction between the exon1/5´ SS and snRNA U1 within this region is needed for Prp4 independence. It also indicates the influence of the interaction with the pseudouridine which changes the helical structure at this position leading to a different binding affinity at position +4 [57,58]. Furthermore, interruption of the Watson-Crick interaction at position +5 leads to Prp4 dependency (Fig 3G and S2 Fig, *res1´-15* and *res1´-16*). However, interruption of base-pairing at position +6 only becomes relevant, if there are no Watson-Crick interactions at positions +3 and +4 of the 5’ SS. In this case the intron is not recognized and therefore retained even in presence of Prp4 kinase activity. This shows that only three and intermittent base-pair interactions in the 5´ SS with snRNA U1 are insufficient for intron recognition (Fig 3H).

The snRNA U1 not only interacts with the 5´ SS of the intron, but also with the last three nucleotides of the exon 1 [44]. In general, the 5’ SS consensus sequence differs from the exon 1 sequences in that the 5’ intron sequences are much more highly conserved, whereas the three nucleotides of the exon 1 sequences are much more variable (Fig 2C). For example, for three introns in the same gene, it would be uncommon for the three nucleotides upstream of the 5’ SSs to be identical. Therefore, this region was also examined in this study regarding Prp4 dependency. As we have shown, if there is no interaction within exon 1 these introns are spliced Prp4-dependently (Fig 3I, *res1’-13* and Fig 4F, *ppk8’-5*). The same results were obtained when an interaction only at position -3 was present (Fig 3I, *res1’-12* and Fig 4F, *ppk8’-6*). Interestingly, formation of hydrogen bonds at positions -1 or -2 could stabilise the interaction between snRNA U1 and exon1/5´ SS, leading to Prp4 independence (Fig 3I, *res1’-10* and *res1’-11* and Fig 4F, *ppk8’-7* and *ppk8’-8*). This rule could also be confirmed by the two introns of the wildtype gene *mrp17* (Fig 2D). In this case, intron I has only one possible Watson-Crick base-pairing at position -3 (CCA/GUAAGU) and is Prp4-dependent (compare with Fig 3I, *res1´-13*). On the contrary, intron II displays one Watson-Crick interaction at position -2 and one wobble base-pairing at position -3 (UAA/GUAUGU) which leads to Prp4 independence (compare with Fig 4F, *ppk8´-7*). Probably, stabilising this interaction within the exon 1 helps to determine the proper site where the first transesterification reaction occurs.

Additionally, mutations in the bs were combined with a strong or weak exon1/5´ SS which had different effects on intron recognition and splicing efficiency (Fig 5). When combined with a weak exon1/5´ SS, mutations within the bs lead to intron retention in nearly all cases even without inhibition of Prp4 kinase. Therefore, the accumulation time course of pre-mRNA after kinase inhibition reflects an additive effect (Fig 5B–5F, *res1´-A-E*). In combination with a strong exon1/5´ SS the mutations in the bs showed different effects on splicing. The change of the branch point is almost invariable and therefore leads to intron retention even if no kinase inhibitor was added (Fig 5B). The mutation at position 2 also resulted in complete intron retention even if no kinase inhibitor was added (Fig 5D, *res1´-2C*). Most interestingly, this position is 100% conserved in all *S*. *pombe* introns (Fig 2C). The third position in the bs is the most degenerate, and can be occupied by any nucleotide (Fig 2C); this position interacts with a pseudouridinylated nucleotide at the end of snRNA U2 which is responsible for bulging out the branch point [54,59] (Fig 5A). The mutation of this position leads to a clear Prp4-dependent intron (Fig 5C, *res1´-2B*). On the contrary, the interruption of possible base-pairing at positions 1 and 5 seems to play a minor role since splicing is still independent on Prp4 kinase activity, although after inhibition of the kinase splicing efficiency decreased slightly in both cases (Fig 5E, *res1’-2D* and 5F, *res1’-2E*).

Although the underlying rules seem to be very complex, it is obvious that Prp4-dependent introns are distinguished from Prp4-independent introns by their reduced potential for hydrogen bonding between the exon1/5’ SS region and snRNA U1 or between the bs and snRNA U2. A complementary interaction between the exon1/5´ SS region and snRNA U1 serves as a default state, marking the structure for the first transesterification reaction in the pre-spliceosome. A similar structural marking for proper hydrogen bonding is also associated with the bs-U2 interaction thereby determining the nucleotides where the second transesterification reaction will occur. So far, we can only speculate here about the consequences of the phosphorylation of Prp1 and Srp2 by Prp4 kinase and propose that the phosphorylation by Prp4 might play a role in stabilising the interaction between the SSs and the snRNAs allowing time in concert with the other proteins to display the proper intron borders for the transesterification reactions. Prp1 is a spliceosomal protein operating at the level of precatalytic spliceosomes; therefore, it is reasonable to speculate that phosphorylation of Prp1 could be involved in adjusting a precatalytic spliceosome on introns displaying weak SSs until proper hydrogen bonds between the pre-mRNA and snRNA U1 and U2 are formed. Phosphorylation of Srp2 by Prp4 might play a similar role, helping precatalytic spliceosomes with the recognition of introns and thereby stabilising their interaction with weak exon1/5’ SSs and weak branch sequences. Indeed, it is known for mammalia that the SR protein hsSRSF1 binds to the pre-mRNA and subsequent phosphorylation affects its interaction with a protein of the U1 particle [23]. In *S*. *pombe* it has been shown that Srp2 interacts with spUaf2 which binds to the 3´ SS [60]. It seems likely that it also takes part in exon1/5´ SS recognition. However, there are still open several questions regarding this mechanism. Particularly, we hope to identify further components involved and thereby advance our knowledge about the function of Prp4 kinase in the splicing of introns displaying weak SSs.

## Materials and Methods

### Yeast strains and growth conditions

The standard genetic and molecular techniques used in this study were described previously [61,62]. All strains used in this study are listed in S1 Table.

### Construction of an analogue-sensitive Prp4 kinase

The analogue-sensitive *prp4-as2* kinase allele was generated by introducing a point mutation into the kinase domain at position 238; this mutation changes the gatekeeper phenylalanine residue to alanine [63,64]. The *prp4-as2* allele was fused to the *kanMx*^R^ gene using the pRS426 vector in yeast recombinational cloning [65,66]. The resultant construct was used to produce a PCR fragment, containing the *prp4-as2* kinase allele and the resistance marker, which was transformed into the wild-type strain L972. Growing colonies were selected on plates containing geneticin. Proper replacement of the *prp4* locus on chromosome III was confirmed in geneticin-resistant transformants by PCR using the appropriate primers [67,68].

### Construction of the reporter genes *res1´* and *ppk8´*

To construct reporter genes, *res1´* and *ppk8´* were fused to the thiamine-repressible *nmt1-8* promoter by cloning the open reading frames into vector pML81HA [69,70]. Both open reading frames contain a frameshift between the HA-tag and the ATG, creating a stop codon at the beginning of the gene; this is intended to exclude the influence of additional Res1 molecules on cell-cycle regulation. To distinguish between the original *res1* and *ppk8* transcripts and the *res1´* and *ppk8´* transcripts at the *leu1* locus by RT-PCR, a *Hin*dIII restriction site was introduced into exon 1 of both genes (Figs 3A and 4A). The forward primers in each pair (res1_Mut_F, ppk8_Mut_F) detect the *Hin*dIII site.

### Reverse transcription-PCR

To determine the pre-mRNA splicing patterns of intron-containing genes, RNA was extracted from whole-cell extracts. For RT-PCR, RNA was treated with RQ1 RNase-free DNase (Promega) to eliminate possible DNA contaminants. Five micrograms of RNA was treated with 2.5 U RQ1 DNase in reaction buffer containing 40 mM Tris–HCl (pH 8.0), 10 mM MgSO_4_, and 10 mM CaCl_2_. After incubation for 10 min at 37°C, RQ1 DNase was inactivated by the addition of 2 mM EGTA (pH 8.0) and heating to 80°C for 10 min. RNA was reverse transcribed and cDNA was amplified using *Tth* reverse transcriptase (Roboklon, EURX). RNA was incubated for 3 min at the calculated annealing temperature in the presence of 1× *Tth* RT Buffer, 0.25 mM dNTP mix, 20 pmol reverse primer, 2 mM MnCl_2_, and 0.25 U/ml *Tth* RT, followed by incubation at 70°C for 25 min. PCR mix containing 1× PCR-Buffer Pol A, 80 pmol reverse primer, 100 pmol forward primer, and 2 mM MgCl_2_ was then added. cDNA was amplified with 28−45 cycles of 94°C for 30 s, 53−60°C for 30 s, and at 72°C for 60 s. The primer sequences are provided in S2 Table. PCR products were resolved on 2% agarose gels.

### Sequencing of Prp4_as2_ sample libraries

Directional mRNA sequencing libraries were prepared by combining the Illumina TruSeq mRNA-Seq and Illumina TruSeq small RNA protocols. Briefly, mRNA selection was performed using 4 μg of total RNA and oligo-dT beads, as described in the low throughput protocol for Illumina TruSeq RNA sample preparation. The mRNA was subjected to fragmentation at 94°C, treated with Antarctic Phosphatase (NEB) and T4 polynucleotide kinase (NEB), and then purified using RNeasy MinElute spin columns (Qiagen). TruSeq indexed RNA adapters were ligated to the RNA and further processing, including 11 cycles of PCR for library amplification, was performed as described in the Illumina v1.5 small RNA protocol. Finally, fragments corresponding to an insert size of 250–500 nt were selected on a 6% Novex TBE gel (Invitrogen). After elution from the gel slice, library quality was confirmed using a DNA 1000 Bioanalyzer chip on an Agilent 2100 Bioanalyzer and. Sensitive quantitation was performed using a KAPA Library Quantification Kit (Kapa Biosystems). Five indexed libraries were pooled and run in each HiSeq lane using Illumina HiSeq v3 sequencing chemistry. Base calling was performed using the Illumina pipeline software version 1.8.1 (within HCS 1.4.8). Adapters used during library preparation were removed from reads using the TagDust tool [71]; approximately 1% of the initial reads were removed in this way. Reads were mapped to the *S*. *pombe* genome using the TopHat software (v2.0.5) [72]. The TopHat alignment was performed using the annotation defined in the Ensembl database (version ASM294 v1.15), taking into account the orientation of the reads. The percentage of mapped reads was approximately 95% for all samples. Finally, the unequivocally mapped reads (85–89% of the initial reads) were selected for further analysis. The average coverages for the exonic and intronic spaces were determined considering only genomic elements longer than 30 bp. This information was then plotted as three different graphs showing the coverage of 5’ exons, introns, and 3’ exons. Exons that were defined simultaneously as the 5’ exon for one intron and the 3’ exon for another intron were considered twice. Finally, the coverage was calculated by normalization to the total number of mapped reads using BEDTools [73]. Plots were generated with custom R scripts.

The RNA-seq data have been deposited in NCBI's Gene Expression Omnibus and are accessible through GEO Series accession number GSE75517 (https://www.ncbi.nlm.nih.gov/geo/query/acc.cgi?acc=GSE75517)

### Detection of intron retention

For each intron, the number of split and unsplit reads that mapped to the splice junction (defined as the three bases around the 5’ exon junction) were counted, and the base-2 logarithm of the ratio between the two values was calculated. This value is called the Relative Splicing Efficiency Index (RSEI). If the RSEI was positive, then more reads indicated spliced mRNA than unspliced pre-mRNA. On the other hand, a negative RSEI indicates more unspliced than spliced RNA. Only intron sequences with more than ten reads for each sample were used for further analysis. This approach identified 2557 Prp4-independent introns (i.e., those with a positive RSEI irrespective of Prp4 inhibition) and 1008 Prp4-dependent introns (i.e., those with a negative RSEI in the presence of kinase inhibitor).

## Supporting Information

S1 Table *Schizosaccharomyces pombe* strains used in this study. (DOCX)

S2 Table RT-PCR primers used in this study. (DOCX)

S1 Fig Prp4_as2_ kinase and its inhibition by 1NM-PP1 after the *res1* gene is replaced by the intronless version *res1Δintron*. (A) A strain with the genotype *h*^*-s*^ *prp4-as2 res1Δintron* was grown at 30°C to early log-phase. The inhibitor 1NM-PP1 was then added to the culture medium (0 hours, arrow, **↓**) at a final concentration of 10 μM. Growth of the culture was monitored by counting the number of cells/ml **(**squares) relative to a culture grown in the absence of the inhibitor (circles). (B) DNA content analysis (in units of C) of *prp4-as2* cells immediately before (-Inh) and at the indicated times after the addition of 1NM-PP1 (+).
(TIF)

S2 Fig The Prp4 kinase dependence of the *res1* intron can be changed by mutations in the exon1/5’ splice site. RT-PCR analysis in the absence (-Inh) and presence (+Inh) of inhibitor at the indicated times. H_2_O, negative control without template. The scheme on the left side of the image shows the details of the interactions between exon1/5’ SS and snRNA U1. Small letters indicate the mutations in *res1’* exon1/5’ SS; the corresponding alleles were named as indicated. |, Watson-Crick base-pairing; Ψ, Pseudouridine; ϕ, wobble base-pairing Ψ-A. Asterisks indicate the expected position of fragments if the introns are not spliced out. The numbers on the left side of the images represent the sizes of the DNA fragments (bp). M, DNA size marker.
(TIF)

S3 Fig Prp4 kinase-independent and Prp4 kinase-dependent introns remain what they are, if inserted into the naturally intronless *ura4* gene. The intron II of the *ade2* gene (383 bp; RSEI +2,28; Prp4 kinase-independent) was inserted into the naturally intronless *ura4* gene (strain 933) as well as the intron of the *res1* gene (127 bp; RSEI -1,36; Prp4 kinase-dependent; strain 930). RT-PCR analysis in the absence (-Inh) and presence (+Inh) of inhibitor at the indicated times. H_2_O, negative control without template. The numbers on the right side of the image represent the sizes of the RT-PCR fragments (bp). Asterisks indicate the expected position of fragments if the introns are not spliced out. The numbers on the left side of the images represent the sizes of the DNA fragments (bp). M, DNA size marker.
(TIF)
