# Supplementary material for: Prp4 Kinase Grants the License to Splice: Control of Weak Splice Sites during Spliceosome Activation
Source: PLoS Genet. 2016 Jan 5;12(1):e1005768. doi: 10.1371/journal.pgen.1005768 (PMC4701394; doi:10.1371/journal.pgen.1005768)
Supplement: S1 Table — (DOCX) [file pgen.1005768.s001.docx]

**S1 Table. *Schizosaccharomyces pombe* strains used in this study**

| Strain | Genotype |
| --- | --- |
| 801 | *h^-S^ prp4 int::prp4-as2-kan^R^* |
| 930 | *h^-S^ prp4 int::prp4-as2-kan^R^ ura4-D18 int::ura4+ade2-II-Intron (383 bp)* |
| 933 | *h^-S^ prp4 int::prp4-as2-kan^R^ ura4-D18 int::ura4+res1-Intron (127 bp)* |
| 971 | *h^-S^ prp4 int::prp4-as2-kan^R^ leu1-32 int::pML81HA-res1´-1 ura4-D18* |
| 978 | *h^-S^ prp4 int::prp4-as2-kan^R^ res1 int::res1∆intron-nat^R^* |
| 991 | *h^-S^ prp4 int::prp4-as2-kan^R^ leu1-32 int::pML81HA-res1´ ura4-D18* |
| 992 | *h^-S^ prp4 int::prp4-as2-kan^R^ leu1-32 int::pML81HA-res1´-A ura4-D18* |
| 993 | *h^-S^ prp4 int::prp4-as2-kan^R^ leu1-32 int::pML81HA-res1´-B ura4-D18* |
| 995 | *h^-S^ prp4 int::prp4-as2-kan^R^ leu1-32 int::pML81HA-res1´-E ura4-D18* |
| 996 | *h^-S^ prp4 int::prp4-as2-kan^R^ leu1-32 int::pML81HA-res1´-D ura4-D18* |
| 997 | *h^-S^ prp4 int::prp4-as2-kan^R^ leu1-32 int::pML81HA-res1´-C ura4-D18* |
| 998 | *h^-S^ prp4 int::prp4-as2-kan^R^ leu1-32 int::pML81HA-res1´-2A ura4-D18* |
| 999 | *h^-S^ prp4 int::prp4-as2-kan^R^ leu1-32 int::pML81HA-res1´-2D ura4-D18* |
| 1000 | *h^-S^ prp4 int::prp4-as2-kan^R^ leu1-32 int::pML81HA-res1´-2C ura4-D18* |
| 1001 | *h^-S^ prp4 int::prp4-as2-kan^R^ leu1-32 int::pML81HA-res1´-2E ura4-D18* |
| 1006 | *h^-S^ prp4 int::prp4-as2-kan^R^ leu1-32 int::pML81HA-res1´-2B ura4-D18* |
| 1018 | *h^-S^ prp4 int::prp4-as2-kan^R^ leu1-32 int::pML81HA-res1´-8 ura4-D18* |
| 1019 | *h^-S^ prp4 int::prp4-as2-kan^R^ leu1-32 int::pML81HA-res1´-10 ura4-D18* |
| 1020 | *h^-S^ prp4 int::prp4-as2-kan^R^ leu1-32 int::pML81HA-res1´-11 ura4-D18* |
| 1021 | *h^-S^ prp4 int::prp4-as2-kan^R^ leu1-32 int::pML81HA-res1´-13 ura4-D18* |
| 1022 | *h^-S^ prp4 int::prp4-as2-kan^R^ leu1-32 int::pML81HA-res1´-18 ura4-D18* |
| 1023 | *h^-S^ prp4 int::prp4-as2-kan^R^ leu1-32 int::pML81HA-res1´-7 ura4-D18* |
| 1024 | *h^-S^ prp4 int::prp4-as2-kan^R^ leu1-32 int::pML81HA-res1´-17 ura4-D18* |
| 1025 | *h^-S^ prp4 int::prp4-as2-kan^R^ leu1-32 int::pML81HA-res1´-9 ura4-D18* |
| 1026 | *h^-S^ prp4 int::prp4-as2-kan^R^ leu1-32 int::pML81HA-res1´-1 ura4-D18* |
| 1028 | *h^-S^ prp4 int::prp4-as2-kan^R^ leu1-32 int::pML81HA-res1´-12 ura4-D18* |
| 1029 | *h^-S^ prp4 int::prp4-as2-kan^R^ leu1-32 int::pML81HA-res1´-15 ura4-D18* |
| 1030 | *h^-S^ prp4 int::prp4-as2-kan^R^ leu1-32 int::pML81HA-res1´-6 ura4-D18* |
| 1031 | *h^-S^ prp4 int::prp4-as2-kan^R^ leu1-32 int::pML81HA-res1´-16 ura4-D18* |
| 1032 | *h^-S^ prp4 int::prp4-as2-kan^R^ leu1-32 int::pML81HA-res1´-4 ura4-D18* |
| 1033 | *h^-S^ prp4 int::prp4-as2-kan^R^ leu1-32 int::pML81HA-res1´-5 ura4-D18* |
| 1034 | *h^-S^ prp4 int::prp4-as2-kan^R^ leu1-32 int::pML81HA-ppk8´ ura4-D18* |
| 1037 | *h^-S^ prp4 int::prp4-as2-kan^R^ leu1-32 int::pML81HA-ppk8´-3 ura4-D18* |
| 1038 | *h^-S^ prp4 int::prp4-as2-kan^R^ leu1-32 int::pML81HA-ppk8´-4 ura4-D18* |
| 1039 | *h^-S^ prp4 int::prp4-as2-kan^R^ leu1-32 int::pML81HA-ppk8´-1 ura4-D18* |
| 1042 | *h^-S^ prp4 int::prp4-as2-kan^R^ leu1-32 int::pML81HA-ppk8´-2 ura4-D18* |
| 1048 | *h^-S^ prp4 int::prp4-as2-kan^R^ leu1-32 int::pML81HA-res1´-3 ura4-D18* |
| 1049 | *h^-S^ prp4 int::prp4-as2-kan^R^ leu1-32 int::pML81HA-res1´-11 ura4-D18* |
| 1050 | *h^-S^ prp4 int::prp4-as2-kan^R^ leu1-32 int::pML81HA-ppk8´-5 ura4-D18* |
| 1051 | *h^-S^ prp4 int::prp4-as2-kan^R^ leu1-32 int::pML81HA-ppk8´-8 ura4-D18* |
| 1052 | *h^-S^ prp4 int::prp4-as2-kan^R^ leu1-32 int::pML81HA-ppk8´-7 ura4-D18* |
| 1053 | *h^-S^ prp4 int::prp4-as2-kan^R^ leu1-32 int::pML81HA-ppk8´-6 ura4-D18* |
