# Supplementary material for: Prp4 Kinase Grants the License to Splice: Control of Weak Splice Sites during Spliceosome Activation
Source: PLoS Genet. 2016 Jan 5;12(1):e1005768. doi: 10.1371/journal.pgen.1005768 (PMC4701394; doi:10.1371/journal.pgen.1005768)
Supplement: S2 Table — (DOCX) [file pgen.1005768.s002.docx]

**S2 Table. RT-PCR primers used in this study**

| **Primer** | **Sequence (5´-3´)** |
| --- | --- |
| cdc2_E1_F | TACCGCTAGGTTGCTTCTTTT |
| cdc2_E3_R | GCCCCAGTTTCTGAAATTCG |
| cdc2_E3_F | CTTGCGAGGTCATTTGGTGT |
| cdc2_E5_R | TGGGCAGGGTCATAAACAAG |
| mrp17_E1_F | CTAGGCCAGCTGCAAATG |
| mrp17_E2_R | CTGTGGGGTTACTGTAAAAGG |
| mrp17_E2_F | GGTGTGGTCGTTGATGTTG |
| mrp17_E3_R | GATAATTGGACAGCTCTGCG |
| ppk8_Mut_F | CGGAAGAGAACTCTAATAATTTATTTTGCAA |
| ppk8_WT_F | CGGAAGAGAACTCTAATAATTTATTTTGCAC |
| ppk8_E2_R | CGCTCTCTTCACTCTCAATGG |
| rbp5_E1_F | ATATATGAATTCTTGGAAGACAGCTCATCAATTAGTC |
| rbp5_E2_R | TATATAGAGCTCGAATTGATCCAACGTCAAATCTAAT |
| rbp5_E2_F | ATATATGGATCCTGAATTAGATTTGACGTTGGATCAA |
| rbp5_E3_R | ATATATGAGCTCTAGATTGTGCCTTTATTTGAATCAT |
| res1_E1_F | TTCGCTCAAAAGGGACTTCATG |
| res1_E2_R | AAGGCCGGGAAAATGACTTTTT |
| res1_Mut_F | GAAAAAATTCAAGGTGGTTGTGGAAGC |
| res1_WT_F | GGGACTTCATGAAAAAATTCAAGGTGG |
| res2_E1_F | TTGGTGCGCATGAAAAAGTACA |
| res2_E2_R | CGGCCACGTACAGAAGGCT |
| rpl29_F | ATGGCCAAGTCGAAGAATCATACTA |
| rpl29_R | TTGGTTGCGGCGGAACTTA |
| ura4_F | CTTGGGCTCATATCACAAATTGCC |
| ura4_R | CAGCCCGTCTCCTTTAACATCC |
| tbp1_E1_F | AATTAAGGATCCGGATGCAGAAGTTTCAAAAAATGAAGGTG |
| tbp1_E2_R | TTACGTGAGCTCAGCGCAATAGTTTTGAGATCAAGACG |
| tbp1_E2_F | ATATATGGATCCTGTCGTCTTGATCTCAAAACTATTGCG |
| tbp1_E3_R | TATATAGAGCTCTTGCCACCCAAAACAACCATTTTACC |
| tbp1_E3_F | ATATTAGGATCCCATTGTAGGAAGTTGCGATGTTAAATTTCCA |
| tbp1_E4_R | ATATATGAGCTCCTTTCGCACCAGTTAAAACAATTTTACCA |
