# Supplementary figures and images for: Prp4 Kinase Grants the License to Splice: Control of Weak Splice Sites during Spliceosome Activation
Source: PLoS Genet. 2016 Jan 5;12(1):e1005768. doi: 10.1371/journal.pgen.1005768 (PMC4701394; doi:10.1371/journal.pgen.1005768)

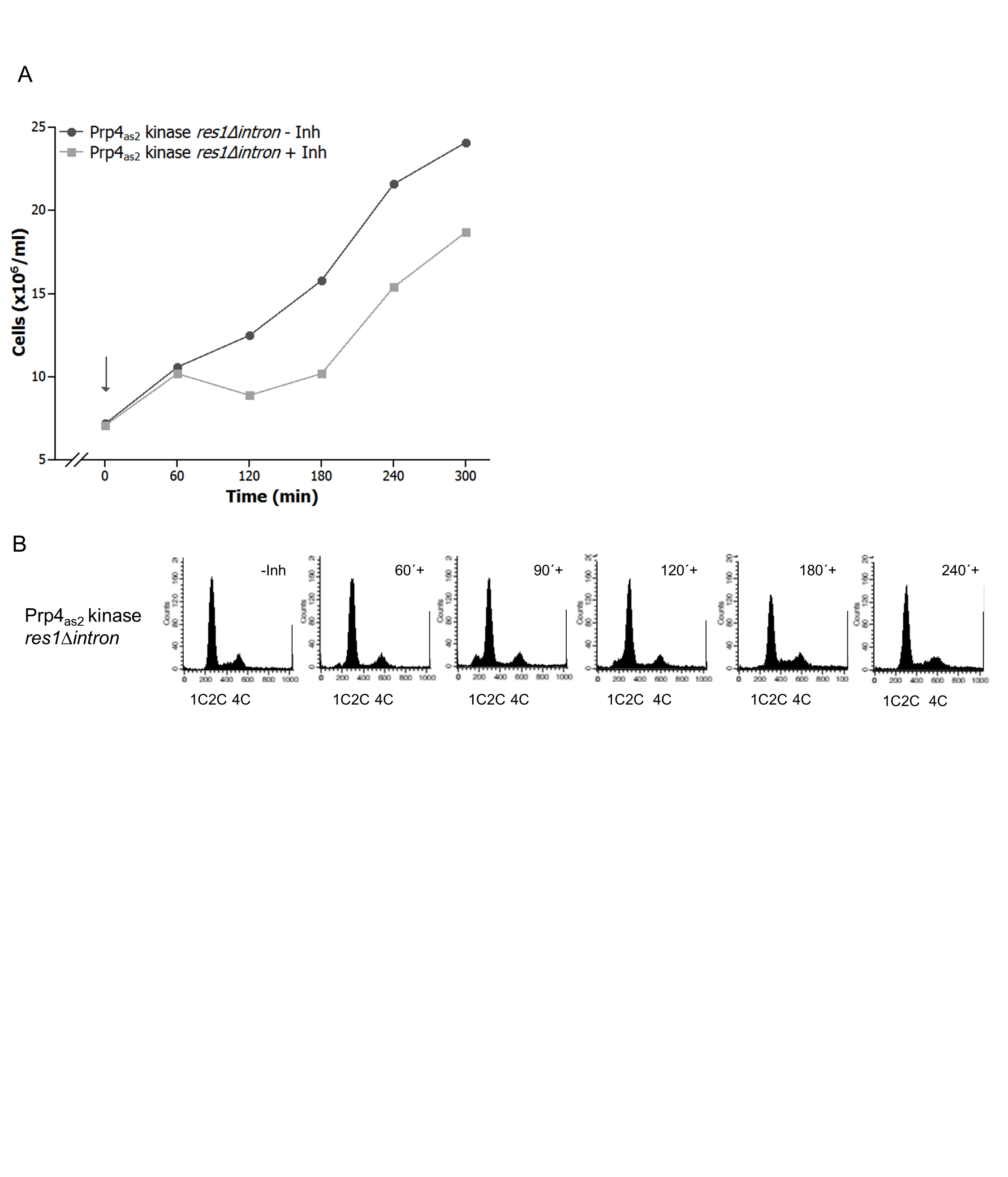

Supplement: S1 Fig — (A) A strain with the genotype h-s prp4-as2 res1Δintron was grown at 30°C to early log-phase. The inhibitor 1NM-PP1 was then added to the culture medium (0 hours, arrow, ↓) at a final concentration of 10 μM. Growth of the culture was monitored by counting the number of cells/ml (squares) relative to a culture grown in the absence of the inhibitor (circles). (B) DNA content analysis (in units of C) of prp4-as2 cells immediately before (-Inh) and at the indicated times after the addition of 1NM-PP1 (+). (TIF) [file pgen.1005768.s003.TIF]

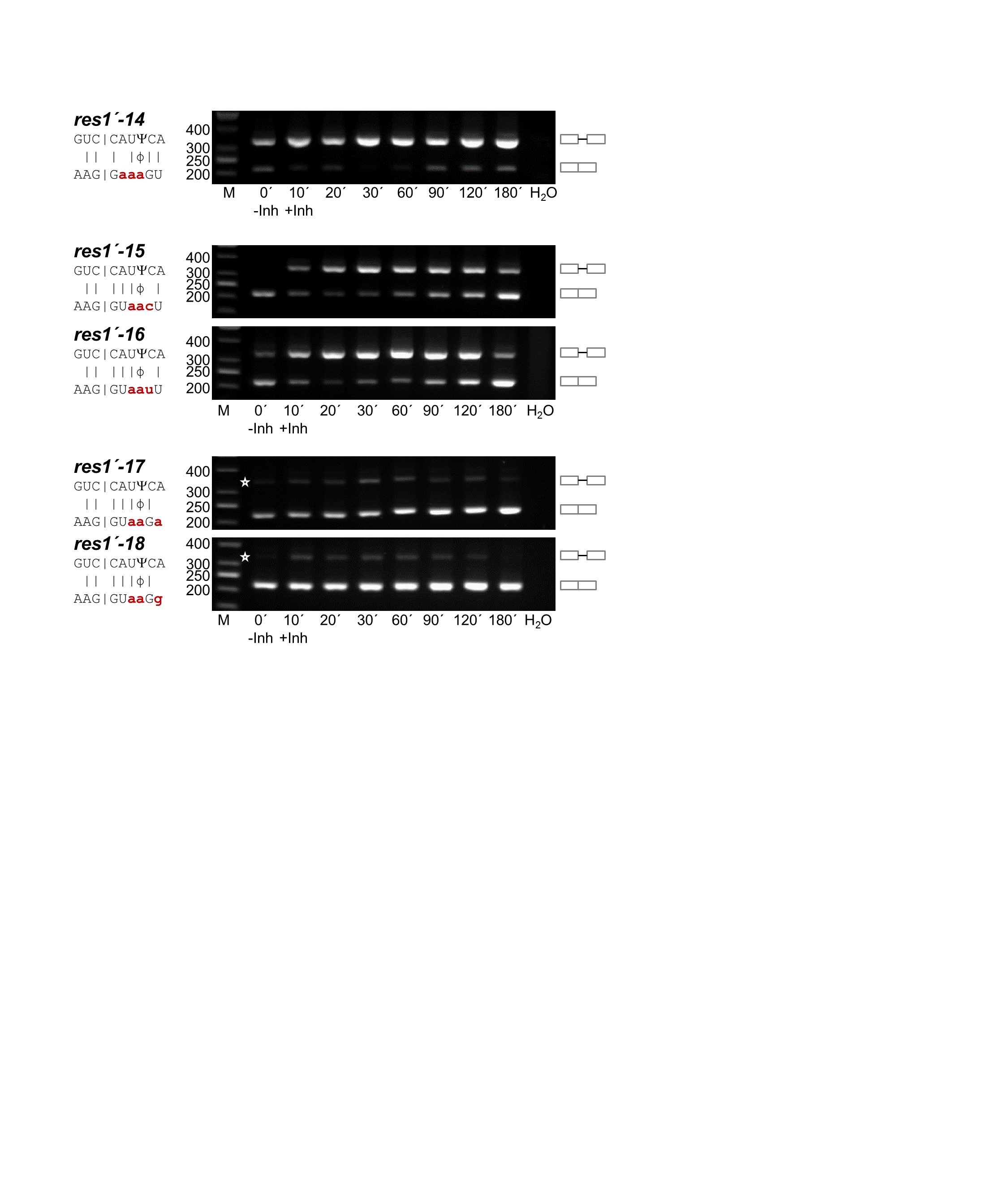

Supplement: S2 Fig — RT-PCR analysis in the absence (-Inh) and presence (+Inh) of inhibitor at the indicated times. H2O, negative control without template. The scheme on the left side of the image shows the details of the interactions between exon1/5’ SS and snRNA U1. Small letters indicate the mutations in res1’ exon1/5’ SS; the corresponding alleles were named as indicated. |, Watson-Crick base-pairing; Ψ, Pseudouridine; ϕ, wobble base-pairing Ψ-A. Asterisks indicate the expected position of fragments if the introns are not spliced out. The numbers on the left side of the images represent the sizes of the DNA fragments (bp). M, DNA size marker. (TIF) [file pgen.1005768.s004.TIF]

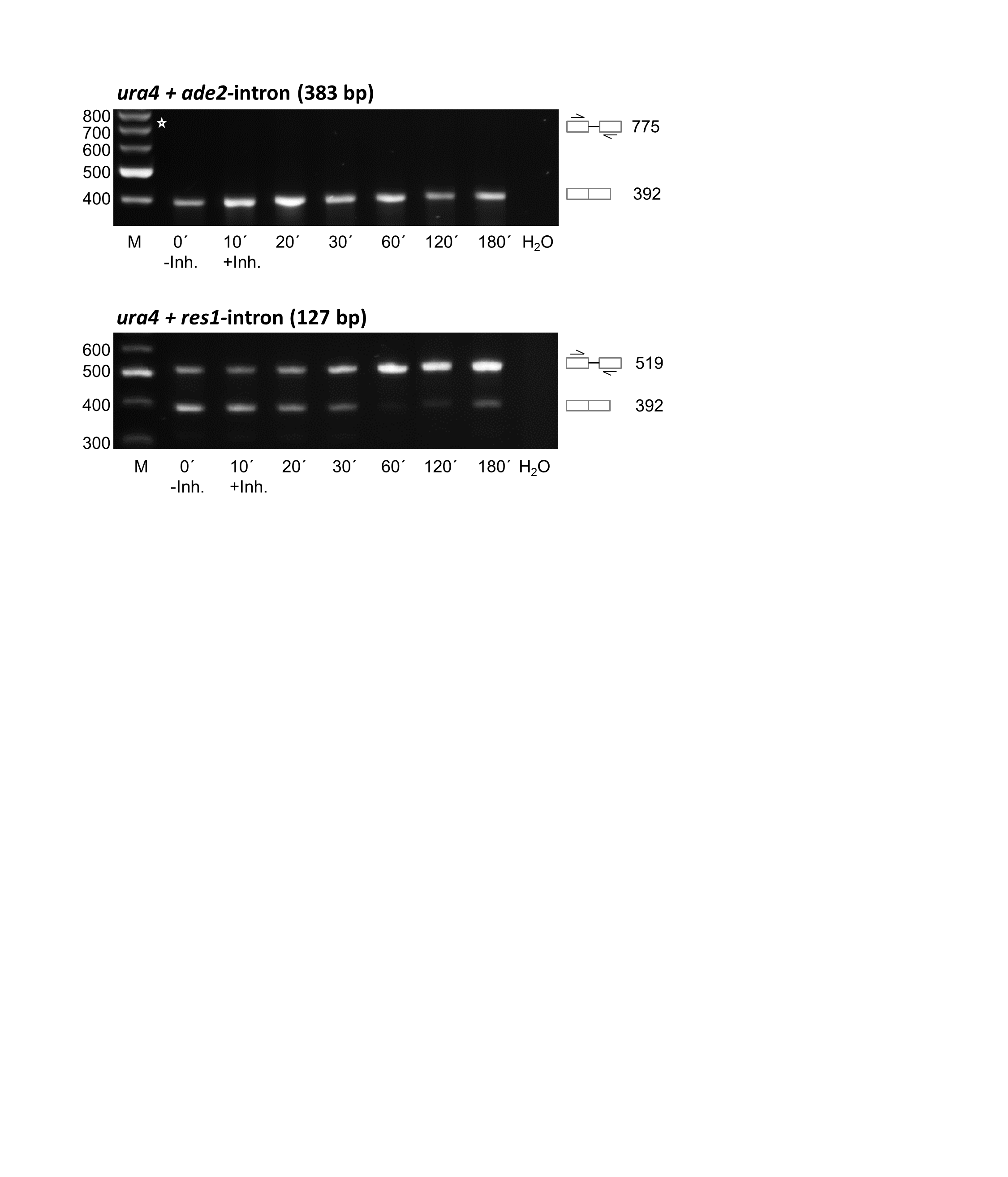

Supplement: S3 Fig — The intron II of the ade2 gene (383 bp; RSEI +2,28; Prp4 kinase-independent) was inserted into the naturally intronless ura4 gene (strain 933) as well as the intron of the res1 gene (127 bp; RSEI -1,36; Prp4 kinase-dependent; strain 930). RT-PCR analysis in the absence (-Inh) and presence (+Inh) of inhibitor at the indicated times. H2O, negative control without template. The numbers on the right side of the image represent the sizes of the RT-PCR fragments (bp). Asterisks indicate the expected position of fragments if the introns are not spliced out. The numbers on the left side of the images represent the sizes of the DNA fragments (bp). M, DNA size marker. (TIF) [file pgen.1005768.s005.TIF]
